# Centering Equity and Fostering Stakeholder Collaboration and Trust—Pillars of the Maternal Health Innovation Program in Maryland

**DOI:** 10.1089/heq.2023.0127

**Published:** 2024-06-27

**Authors:** Andreea A. Creanga, Briana Kramer, Carrie Wolfson, Meighan Mary, Elizabeth M. Stierman, Sarah Clifford, Ada Ezennia, Jane Rhule, Nina Martin, Maxine Vance-Reed, Teneele Bruce, Bonnie DiPietro, Adriane Burgess, Nicole Warren, Shari N. Lawson, Sarah Meyerholz, Kelly Bower

**Affiliations:** ^1^ Department of International Health, Johns Hopkins Bloomberg School of Public Health, Baltimore, Maryland, USA.; ^2^ Department of Gynecology and Obstetrics, Johns Hopkins School of Medicine, Baltimore, Maryland, USA.; ^3^ Maryland Department of Health, Maternal and Child Health Bureau, Baltimore, Maryland, USA.; ^4^ Baltimore Healthy Start, Baltimore, Maryland, USA.; ^5^ Maryland Patient Safety Center, Elkridge, Maryland, USA.; ^6^ Maternal & Women’s Health Branch, Division of Healthy Start and Perinatal Services, Health Resources and Services Administration, Maternal and Child Health Bureau, Rockville, Maryland, USA.; ^7^ Johns Hopkins University School of Nursing, Baltimore, Maryland, USA.

**Keywords:** maternal health, health equity, stakeholders, program implementation, evaluation

## Abstract

**Objective:**

To describe two main pillars of the Maryland Maternal Health Innovation Program (MDMOM): (1) centering equity and (2) fostering broad stakeholder collaboration and trust.

**Methods:**

We summarized MDMOM’s key activities and used severe maternal morbidity (SMM) surveillance and program monitoring data to quantify MDMOM’s work on the two pillars. We developed measures of hospital engagement with MDMOM (participation in quality improvement [QI] activities, participation in check-in meetings, staff involvement) and with other partners (participation in QI activities, representation in state-level groups). We examined Bonferroni-adjusted correlations between these hospital engagement measures and with key hospital characteristics: level of maternity care, annual delivery volume, and SMM rate.

**Results:**

Over 100 national and state organizations and individual stakeholders contributed to our building the MDMOM program and implementing key activities centering equity: hospital-based SMM surveillance in 20 of Maryland’s 32 hospitals; almost 5,000 trainings offered to perinatal health care providers; two telemedicine/telehealth interventions; training of home visitors and community-based organization staff. Birthing hospitals represent MDMOM’s main implementation partners. The strength of their participation in MDMOM QI activities is positively correlated to their participation in check-in meetings and with the degree of involvement by physicians in such activities. Higher engagement in MDMOM QI activities is also positively correlated to hospitals’ participation in other state-level maternal health initiatives or groups.

**Conclusion:**

Our experience with the MDMOM program demonstrates that an equity focus and broad stakeholder collaboration building strong relationships and providing implementation support can lead to high levels of engagement in innovative maternal health interventions.

## Introduction

The United States is in a maternal health crisis.^1,2^ Maternal mortality, a key marker of population health and health inequalities, increased continuously over the past 30 years^2,3^ and reached its highest level (32.6 deaths per 100,000 live births) during the COVID-19 pandemic in 2021.^4^ For every 100 deliveries, 2–3 persons experience pregnancy-related complications that result in severe health consequences.^5–8^ Striking, persistent, and well-documented racial disparities exist in maternal health outcomes, with Black women being 1.5–2 times more likely to experience^6–8^ and to die^1,2,9^ from severe maternal morbidity (SMM) than White women. The COVID-19 pandemic exacerbated social and structural factors that shape health behaviors, access to health care, and interactions with medical professionals that long contributed to these disparities.^4,10^ Racism, widely recognized as a key determinant of health status in the United States,^11^ together with other cultural and medical implicit biases among health care professionals play out dramatically in maternal health.^11,12^ There is widespread recognition that these problems need to be addressed. Federal agencies aim to do so through new programs, one of which is Health Resources and Services Administration (HRSA)’s Maternal Health Innovation Program, currently funding demonstration projects in 17 US states.^13^

In September 2019, Maryland received such funding and established the Maryland Maternal Health Innovation program (MDMOM),^14^ which aims to improve maternal health in the state through innovations in data use and service delivery, and changes in hospital culture and operations to focus on health equity. MDMOM has four main components: SMM surveillance and review, which in tandem with the state’s long-standing maternal mortality reviews represent the two main sources of maternal outcome data in the state; a Hospital Equity Initiative and two Telehealth Initiatives, all implemented in collaboration with the state’s 32 birthing hospitals; and the Enhanced Maternal and Postpartum Warning signs Education and Recognition (EMPOWER) Moms Initiative, which supports home-visiting programs and community-based organizations through a community of practice network. Working to center equity in all these efforts, MDMOM builds maternal health workforce capacity through partnership, training, facilitation of peer learning, scheduled and on-demand technical assistance, and a resource library. In addition, to foster collaboration and learning among diverse stakeholders and accelerate implementation of evidence-informed approaches advancing equitable maternal health outcomes, MDMOM established the first statewide Maternal Health Task Force,^15^ with broad membership, coordination of which was transferred to the Maryland Department of Health (MDH) in the fall of 2020.

The purpose of this article is to describe two main pillars of the MDMOM Program: (1) centering equity and (2) fostering broad stakeholder collaboration and trust. We highlight activities aimed at developing new data and quality improvement (QI) initiatives, building workforce awareness and skills to mitigate implicit biases, and increasing access to equitable maternal health care.

## Methods

We first summarized information about the development and implementation of the four MDMOM components to highlight work on the two program pillars of interest. We used data from different program sources to quantify the extent to which MDMOM programming centers equity and fosters collaboration with stakeholders. SMM surveillance data collected between July 1, 2020, and December 31, 2022, and reviewed by multidisciplinary hospital teams were used to show how data on all SMM rates, causes, and preventability are stratified by race ethnicity and shared as such with participating hospitals; detailed surveillance methodology is presented elsewhere.^16–18^ Information collected during regular, usually monthly, check-in meetings with hospitals between October 1, and April 15, 2023 was analyzed using Watkins’ rapid analysis technique.^19^ Relevant quotes depicting the acceptability, adoption, and results of key program activities are presented.

With three of four MDMOM components being hospital based and the fourth sharing resources with hospitals, we developed measures of hospital engagement with MDMOM and with other state partners (Supplementary Table S1). Using program monitoring data collected by April 1, 2023, hospitals’ participation in MDMOM was quantified as the sum of MDMOM QI activities in which the hospital is engaged: any of the trainings offered through the Equity Initiative (range 0–4); participation in the Telehealth Initiative for preeclampsia (range 0–3; coded as no interest = 0; only interest but no activity = 1; distributing BP cuffs or referring to Optum Homecare for 24/7 nursing surveillance = 2; distributing BP cuffs and referring to Optum Homecare = 3); participation in SMM surveillance (range 0–3; coded as no interest = 0; only interest but no activity = 1; entering data = 2; timely and complete case data submission, e.g., quarterly or more frequent review of cases = 3); and utilization of EMPOWER Moms materials in hospitals (range 0–2; coded as no utilization = 0; distribution of warning signs handouts = 1; innovative use of materials in hospitals = 2). Overall, the measure of the strength of participation in MDMOM activities ranged from 0 to 12. Participation in MDMOM meetings was coded as follows: no meeting participation = 0; meeting on as needed basis = 1; meeting every 2–3 months = 2; and regularly attending monthly meetings = 3 (range 0–3). Hospital staff involvement in MDMOM activities was also assessed as the number of individual physicians and nurses, respectively, that interacted with MDMOM staff for any program component since October 1, 2022 (i.e., last 6 months).

Participation in B.I.R.T.H. Equity Maryland led by the Maryland Hospital Association (MHA) and Maryland Patient Safety Center (MPSC) Equity Workgroup in 2023 was also measured and coded as 1 if it occurred and 0 if not. In addition, membership lists were obtained from MHA for the hospital Birth Accountability Group; from MDH for the Morbidity, Mortality, and Quality Review Committee; Maternal Mortality Review Team; and from Maternal Health Task Force. Using these lists, we derived a measure that captures whether birthing hospitals are represented in these four state-level groups (range 0–4).

We next examined Bonferroni-adjusted correlations between hospital engagement measures and with the following hospital characteristics: level of maternity care offered (I–IV); 2021 delivery volumes; and MDH-provided SMM rate categories (<50, 50–99.9, 100–299.9, and ≥300 per 10,000 deliveries) derived from hospital discharge data using International Classification of Diseases codes.^5^ Analyses were conducted in Stata 17.

The Institutional Review Board at the Johns Hopkins Bloomberg School of Public Health has approved research protocols for conducting SMM surveillance (IRB#12192) and evaluating the MDMOM program (IRB#13694).

## Results

### SMM Surveillance and Review

All SMM events in pregnant and up to 42-day postpartum patients admitted at participating hospitals are identified and reviewed using three criteria: admission to an intensive/critical care unit; and/or transfusion of ≥4 units of blood products; and/or hospitalization for management of severe COVID-19 infection. In each hospital, trained clinical abstractors review all relevant medical records for each SMM using a standardized, electronic review form. Multidisciplinary hospital-based committees meet regularly to review and discuss all identified SMM events and make recommendations for preventing future events. MDMOM convenes abstractors from all participating facilities for learning and sharing sessions every 6 weeks to promote knowledge sharing; to date, 23 such sessions have been held.

MDMOM implemented hospital-based SMM surveillance and review in July 2020. Notably, it was Maryland HB-837/2020,^20^ for which MDMOM staff provided testimony, that required the MDH in collaboration with MDMOM to study SMM. We started with a pilot in 6 of the 32 birthing hospitals in Maryland,^16,17^ and surveillance now includes 20 hospitals, covering about 70% of births in the state.^18^

**FIG. 1.**
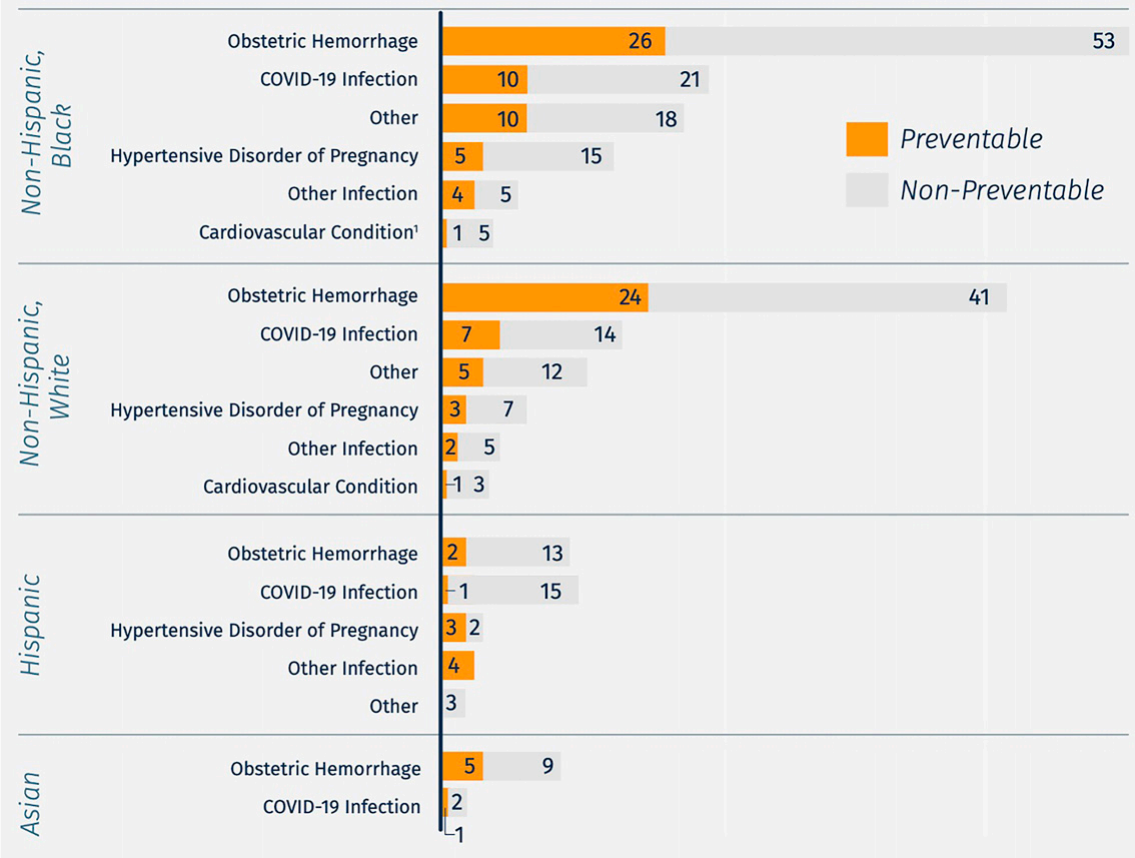
Primary cause of severe maternal morbidity by race-ethnicity and preventability watus: Maryland, 20-hospital severe maternal morbidity surveillance and review (*n* = 374). *Notes*: Reproduced from MDMOM Severe Maternal Morbidity Data Brief. April 2023. Available at: https://mdmom.org/sites/default/files/documents/SMM-MDMOM-Brief-April2023.pdf. Data were collected between July 1, 2020, and December 31, 2022. Surveillance methodology is presented in detail elsewhere.^18^ MDMOM, Maryland Maternal Health Innovation Program.

Analyses of SMM are stratified by race and ethnicity to identify disparities and make recommendations to reduce disparities. Based on 374 SMM events identified from July 1, 2020, to December 31, 2022, the SMM rate was highest for non-Hispanic Black patients, more than double that of non-Hispanic White patients (135.0 vs. 62.9 per 10,000 deliveries, respectively).^18^ Hospital review committees determined that 32% of the SMM events were potentially preventable with changes to provider, system, or patient-level factors.^18^ Group variation in preventability is related to differences in SMM causes by racial-ethnic group (Fig. 1). Findings and recommendations are shared via confidential hospital-specific SMM reports as well as in aggregate via data briefs shared with about 300 stakeholders, including Task Force members.^18^

### Hospital Equity Initiative

This Initiative promotes equity through training and QI activities in maternal health units in Maryland’s 32 birthing hospitals. A series of five CME-eligible trainings was designed (Table 1). Four were bias trainings: a self-paced online implicit bias training; two interactive implicit bias skill building sessions; and a self-paced online training on bias related to substance use disorder (SUD). The fifth training highlights inequitable maternal health outcomes in Maryland and the United States. Except for the first training, developed by Quality Interactions and March of Dimes, all other trainings were developed by MDMOM and expert consultants with input from diverse stakeholders. The two-part implicit bias skill building sessions were developed in collaboration with an expert implicit bias trainer, 8 representatives from community-based organizations serving pregnant people from marginalized communities, and 18 maternal health professionals. The training presenting learnings from SMM/MM reviews was developed in collaboration with a retired obstetrician and epidemiologist with expertise in health disparities research and reviewed by 10 physicians, nurses, and midwives from Maryland hospitals. The SUD training was developed in collaboration with a double-boarded (obstetrics and addiction medicine) physician who is a national expert on SUD and individuals with professional and lived experience, including peer recovery specialists, nurses, physicians, a licensed counselor, and a midwife.

**Table 1.** MDMOM Hospital Equity Initiative Trainings

Title	Description	Learning objectives
*Awareness to Action: Dismantling Bias in Maternal and Infant Health Care*	Self-paced, one-hour, online training developed by the quality interactions and/or March of Dimes^a^	Understand and be able to identify implicit bias, the cognitive basis that informs bias, and its impact on maternity care settings Explain how structural racism has played a key role in shaping U.S. care settings and contributes to implicit biases in patient/provider encounters Recognize your potential for implicit bias and apply strategies, such as the CARES Framework^™^, and practice of cultural humility to effectively mitigate your own implicit biases Recognize how the lessons learned can help create system-wide change to establish a culture of equity that elevates the quality of maternity care
*Mitigating Implicit Bias in Maternal Health Care: Skill Building Session Part 1*	Two interactive, one-hour, skill-building sessions developed and facilitated by MDMOM staff and consultants	Recognize why health care providers are vulnerable to bias Identify common ways that bias impacts patient–provider interactions, patient assessments, provider clinical judgement, and patient outcomes Practice strategies for recognizing and managing bias Commit to practice changes that reduce the impact of bias on patient care and outcomes
*Mitigating Implicit Bias in Maternal Health Care: Skill Building Session Part 2*	Two interactive, one-hour, skill-building sessions developed and facilitated by MDMOM staff and consultants	Identify how bias is communicated nonverbally, paraverbally, and verbally to patients Increase awareness of patient perceptions of clinical interactions Practice communication skills that demonstrate respect in clinical settings Commit to listening and responding to patient concerns
*Learning from Adverse Maternal Events in Maryland*	Self-paced, one-hour, online module or grand rounds developed and presentation by MDMOM staff and consultants	Discuss trends in maternal mortality and severe maternal morbidity in Maryland and the United States Recognize the value of hospital, state and national maternal morbidity, and severe maternal mortality reviews for improving the quality of maternity care Identify key practices in recognition and management strategies for patients with prevalent pregnancy and postpartum complications
*Managing Bias in the Care of Pregnant and Parenting People with Substance Use Disorder*	Self-paced, one-hour, online module or grand rounds developed and presentation by MDMOM staff and consultants	Recognize addiction or SUD as a chronic, treatable condition Describe how stigma and bias create barriers to quality care and equitable outcomes, particularly in pregnancy Recognize how the postpartum period increases vulnerability to morbidity and mortality for people with SUD Identify clinical practices that can be implemented in hospital settings to help reduce bias in the case of people with SUD

*Notes:* ^a^Training series started by offering *Breaking through Implicit Bias in Maternal Health care*, a training co-developed by March of Dimes and Quality Interactions, which was later discontinued.

MDMOM, Maryland Maternal Health Innovation Program; SUD, substance use disorder.

**FIG. 2.**
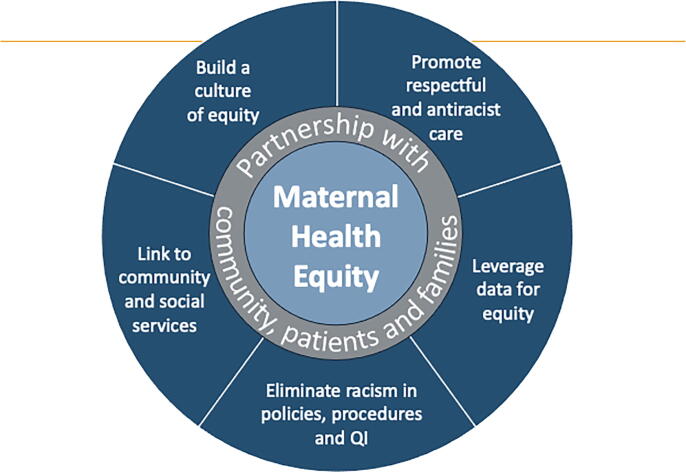
MDMOM maternal health equity toolkit framework. *Notes:* The framework is an adaptation of the Institute for Health care Improvement’s “Framework for Health Care Organizations to Improve Health Equity”.^21^ MDMOM, Maryland Maternal Health Innovation Program; QI, quality improvement.

In addition, a Maternal Health Equity Toolkit was developed to support hospital-based maternal health units to understand racial-ethnic maternal health inequities and implement equity-focused interventions. The Toolkit outlines five priority areas for maternal health equity based on the Institute of Health Care Improvement (IHI) framework for improving health equity: Build a Culture of Equity; Promote Respectful and Antiracist Care; Leverage Data for Equity; Eliminate Racism in Policies, Procedures and Quality Improvement Initiatives; and Link to Community and Social Services (Fig. 2).^21^ The Toolkit also includes a foundational chapter on building essential partnerships with community partners, which introduces principles of equitable collaboration. The content is instructive and practical, developed by MDMOM collaboratively with the leader of a community-based organization, a patient representative from an advocacy organization, and several physician and nurse maternal health equity researchers.

The Initiative was launched in December 2020, benefitting from unprecedented attention to maternal health and health equity,^22^ and a legislative mandate for implicit bias education for perinatal health care providers.^20^ Obstetrics-Gynecology Department leaders in all 32 hospitals were asked to identify a nurse, a physician, and a QI champion to meet regularly with an MDMOM facilitator to plan for and implement the initiative activities.^23^ Prior to initiating the first implicit bias training, we conducted a provider survey to assess knowledge, attitudes, and practices related to implicit bias and other planned MDMOM activities.^12^ After completing this baseline survey, we supported hospital teams to promote MDMOM trainings, aiming to introduce them in a sequential manner using a common learning management platform (Table 2). Almost 5,000 trainings were offered to perinatal health care providers in Maryland by April 1, 2023.

**Table 2.** Planned versus Actual Implementation of Hospital Equity Initiative Activities in Maryland Hospitals

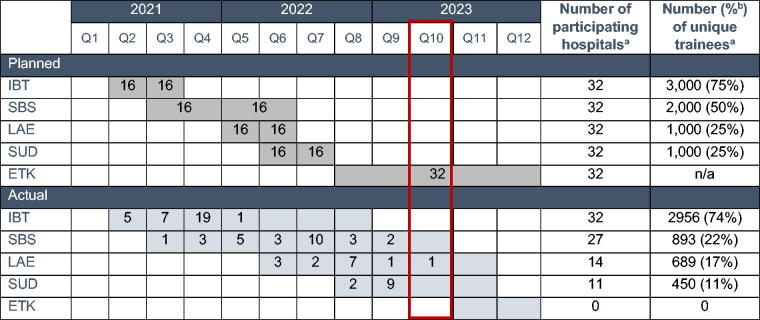

*Notes:* ^a^Actual training data as of April 1, 2023; ^b^Percentage calculated using the estimated 4,000 total maternal health providers across the state per hospital reporting in the fall of 2020. Cells shown in gray denote planned or actual period of implementation. Numbers in cells denote the number of hospitals joining the initiative during the quarter or period. Cells without numbers reflect the continued availability of the activities across all participating hospitals. All trainings will be discontinued by October 1, 2023, whereas implementation of interventions from the Maternal Health Equity Toolkit will continue until September 30, 2024 when the program is set to end or be renewed. The red outline denotes the implementation quarter at the time of article writing.

ETK, Maternal health equity toolkit; IBT, Implicit bias training; LAE, Learnings from adverse maternal events training; SBS, Implicit bias skill-building sessions; SUD, Substance use disorder stigma training.

Implementation of the Equity Initiative was adapted in several ways to maximize hospital and staff participation. Throughout implementation, hospitals faced veritable crises of staffing and acuity, primarily because of the COVID-19 pandemic. Understaffing, burnout, and competing obligations were cited as barriers by every participating hospital. Regular, usually monthly, meetings provided an opportunity to offer relational and implementation support and yielded creative problem solving and adaptation. The chief adaptations related to the implementation timeline. We initially proposed the plan depicted in the top of Table 2 but communicated flexibility at every encounter. Some hospitals delayed implementation, but almost all prioritized implicit bias training, expressing both intrinsic (the topic was timely and valuable) and extrinsic (additional state legislation mandated implicit bias training for health professional licensure beginning in 2022)^24^ motivation. This prioritization resulted in extending the time allowed to introduce and promote our trainings, in some cases for up to a year. Ultimately, the capacity to be responsive to hospitals’ needs enabled high staff participation and furthered the aims of the Initiative by increasing exposure to foundational health equity concepts.

Information reported by hospital colleagues during check-in meetings, albeit prone to social-desirability bias, demonstrate the acceptability and relevance of Equity Initiative trainings in Maryland hospitals (Table 3). Using data from the baseline provider survey, we developed and validated the Bias in Maternal Health Care scale, which contains three subscales that measure bias awareness, bias mitigation practice, and bias mitigation self-efficacy.^12^ We will include this scale in our endline survey to evaluate MDMOM’s Equity Initiative training outcomes in 2024.

**Table 3.** Qualitative Data on MDMOM Activities’ Acceptability, Adoption, and Results

Equity initiative trainings	Telehealth initiative
*IBT training* “[During a difficult interaction with a patient,] a nurse responded in a way that was not mindful. […] Because they'd been doing this training, they had the language to recognize and address the incident […].” *SBS training* “I had staff texting me after [the session] to say it was eye-opening.” “A lot of people were blown away and they weren’t expecting to be.” “I was chatting with a few staff after your 5–6 pm session yesterday and they were saying that the stories made the situations real and that they really think this will make a difference.” *LAE training* “The topic is very important and don’t think staff nurses are familiar with how to define SMM. It is always a good time to learn about this.” “The training is really important and timely so that when we talk about morbidity and mortality people have a shared understanding.” *SUD training* “We want to make this module mandatory for nursing as we've had negative feedback around patients feeling judged or the way messages are being delivered about substance use.” ” […] a patient come in recently to have her fifth baby. She had SUD and was defensive, saying that she knew [we] didn't like to take care of people like her. The nurses who had her said it is their job to take care of people having babies and it made no difference that she had SUD and treated her with a lot of care and respect and the patient was very grateful. The patient reported she'd had very bad experiences with her past four delivery hospitalizations but this was different.” Nurses “feel a sense of urgency” in offering SUD training because it is becoming more of an issue with their patient population and there have been some difficult conversations. […] They said they appreciate any support they can get and think this training might offer that. “It was great and relevant to our patient population. Particularly the video with the moms. It was humanizing.”	“People are excited about this. It is a good opportunity to offer something to our patients with more limited needs.” “I think it has been great, filling a need.” “We are off to a great start with the MDMOM telehealth BP cuff program! We did in-service to all staff throughout the weekend and out of the 12 kits we started with, we only have 5 left. They have been distributed in Triage and before postpartum discharge.” “We think this will be really valuable for our patients.” “We're glad to have the cuffs and get them out.” “A patient came into the office yesterday and was given a BP Cuff. She went home and had a severe range pressure that night and reported it and came into the hospital. Her labs were abnormal and she was induced at term.”
	**Urgent Maternal Warning Signs Handouts**
	“We LOVE the maternal urgent warning signs handouts and give them to every patient in triage and postpartum, it really helps to open up conversations.” “The information is needed and to have this visual reminder would be phenomenal! My vision is to have the signage in all our Labor and Delivery and Mother/Baby rooms and to provide the patients with a copy in their take home folder.” “Really love the warning signs materials, and they check a couple of important regulatory boxes, too!” The Dari-speaking patient was “so surprised and grateful that the materials were available in her language.”
	**Hospital Reports**
	“Looks great! Thank you! This report is helpful for us as we are trying to reduce [our] PPH rates.” “[…] because of the MDMOM SMM data brief, the hospital leadership is putting more resources towards efforts to improve maternal outcomes.” “The report was great! […] the baseline survey results were reviewed during multidisciplinary meeting and provided justification for why we are continuing to push MDMOM initiatives.”

*Notes:* Data are from check-in meetings with hospitals during October–April 2023. Currently, all hospitals are still offering the SBS, LAE, and SUD trainings.

BP, blood pressure; IBT, implicit bias training; LAE, learnings from adverse maternal events training; MDMOM, Maryland Maternal Health Innovation Program; PPH, postpartum hemorrhage; SBS, implicit bias skill-building sessions; SUD, substance use disorder stigma training.

### Telehealth Initiatives

Evidence suggests that telehealth services may have the potential to increase access to care for pregnant and postpartum people,^25^ improve obstetric outcomes,^26^ and significantly decrease racial disparities in postpartum follow-up.^27,28^ MDMOM developed a telehealth portfolio aimed to innovate models of maternal health care and minimize inequities caused by geographic or social barriers to accessing services. Through a landscape analysis of Maryland’s Level I–II hospitals,^29^ MDMOM identified best practices and challenges in implementing perinatal telehealth interventions. A first program launched in January 2022 in five pilot sites in underserved communities, including three Federally Qualified Health Centers (FQHCs), offered home telemedicine consults for high-risk patients and peer-to-peer consultations/telementoring. MDMOM also developed an initiative to address hypertensive disorders in pregnancy, a leading cause of SMM.^18,30^ It encourages home monitoring of blood pressure (BP) for patients at risk of or with severe hypertension in pregnancy/postpartum through: (1) distribution of free BP cuffs and education materials in participating hospitals and (2) referral to Optum Homecare Services for 24/7 telehealth nursing and biometric surveillance of patients at risk or diagnosed with preeclampsia in pregnancy/postpartum. This telehealth initiative emerged from consultations with hospital and community providers, which considered it a potential game changer in the care of patients with severe hypertension in pregnancy in the state. Throughout 2023, across participating hospitals, about six high-risk patients receive a BP cuff everyday.

### EMPOWER Moms

To increase awareness and action on urgent maternal warning signs in Maryland, MDMOM partnered with Baltimore Healthy Start (BHS) to launch the EMPOWER Moms initiative. BHS provides comprehensive and supportive services in communities by conducting door-to-door outreach for pregnant and postpartum women. Upon enrollment, their clients are offered intensive case management, home visiting and medical care coordination, health education, and emergency needs assistance to facilitate positive birth outcomes.

EMPOWER Moms provides home visiting programs and community-based organizations with training and tools to deliver maternal warning signs education and support families in obtaining health care that meets their needs. The initiative brought together 32 programs providing home visiting, community outreach, and center-based services to families in 19 of Maryland’s 24 local jurisdictions. Programs serve predominantly low-income populations, communities of color, and others disproportionately impacted by adverse maternal outcomes. The intervention includes training for staff, monthly Community of Practice meetings, and a toolkit with a 3-minute video, magnet, discussion guide, and illustrated handout—available in 12 languages. Educational materials are based on content developed by U.S. Centers for Disease Control’s Hear Her Campaign^31^ and the Alliance for Innovation on Maternal Health (AIM).^32^ Since its launch in March 2021, 257 home visitors, community health workers, doulas, social workers, educators, and other staff have been trained, and together they have delivered the EMPOWER Moms education to 767 pregnant and 1,477 postpartum clients.

A mass-media campaign with the Hatcher Group promotes public awareness through digital ads, bus and metro displays, and a website. The media campaign has generated 17,630 visits to MDMOM’s maternal warning signs website (https://maternalwarningsigns.org/) and 3,673 views of the 3-minute warning signs video on YouTube (https://www.youtube.com/@mdmomprogram5758).

### Program Stakeholders and Engagement in MDMOM and Other Activities

**FIG. 3.**
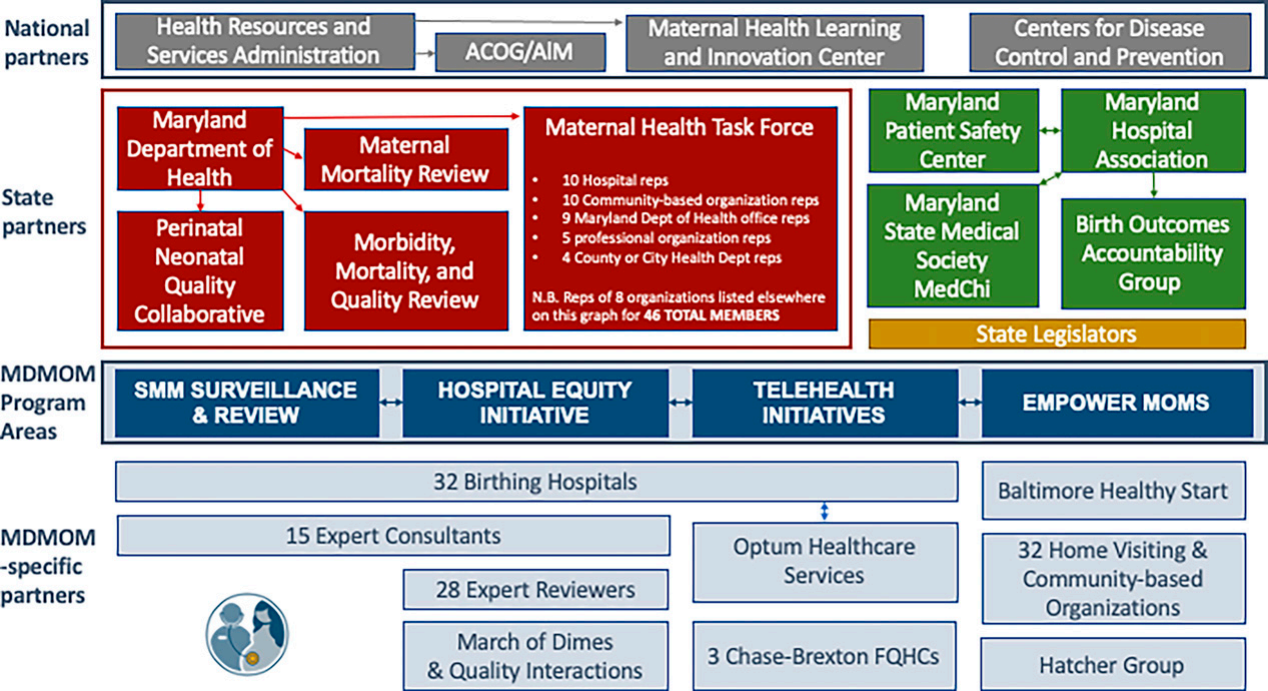
MDMOM program stakeholders. *Notes:* Unidirectional arrows used to represent coordination roles; bidirectional arrows used to represent cooperation and/or shared resources; outlines used to denote close collaborations. ACOG, American College of Obstetricians and Gynecologists; AIM, Alliance for Innovation on Maternal Health; FQHC, Federally Qualified Health Centers; reps, representatives; MDMOM, Maryland Maternal Health Innovation Program.

Over 100 national and state organizations and individual stakeholders contributed to our building the MDMOM program and implementing its activities (Fig. 3). With support from the funding agency (HRSA), MDH, MPSC, and MDMOM established a 46-member Task Force that developed and currently carries out a five-year maternal health Strategic Plan.^33^ MDMOM has only one representative in the Task Force and supports a small contract, renewed annually, for an equity advisor to guide the work of the Task Force. Members of the Task Force as well as representatives from most stakeholders listed in Figure 3 were asked to provide feedback on all trainings and materials developed by the MDMOM program and consulted on program changes and adaptations over time.

**FIG. 4.**
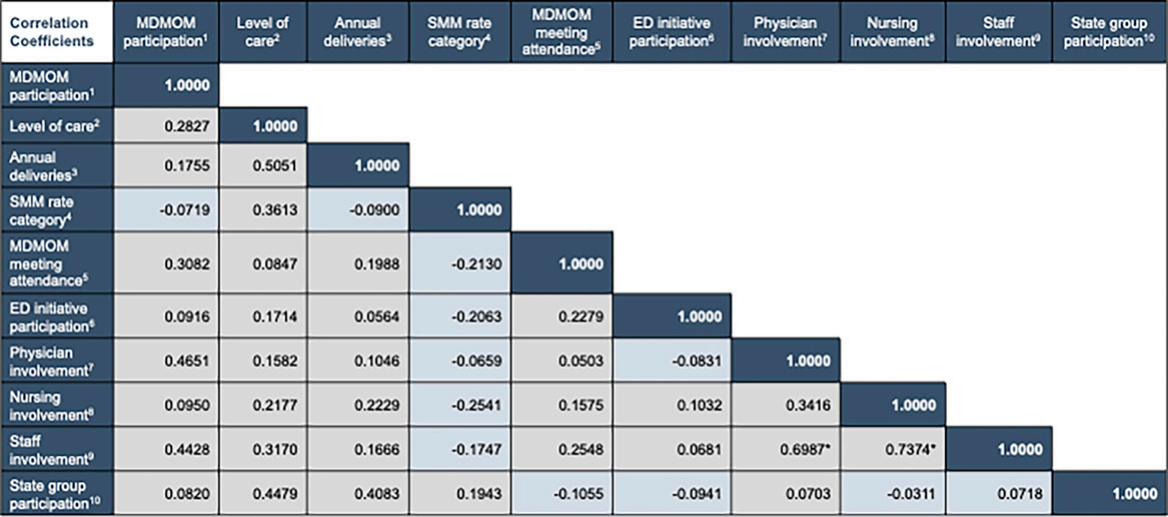
Hospital engagement in MDMOM and other state partner activities (*n* = 32). *Notes:* Shown are pairwise correlation coefficients with Bonferroni-adjusted significance levels at *p* < 0.05 denoted with *.^1^ Calculated as sum of activities and engagement strength ranging from 0 to 12 as per Methods section;^2^ level of maternity care offered by the hospital ranging from I to IV, with Level IV hospitals offering the highest level of care;^3^ 2021 number of deliveries in each hospital;^4^ SMM rate categories ranging from 1 (<50 per 10,000 delivery hospitalizations) to 4 (>300 per 10,000 deliveries) in each hospital;^5^ Ranging from 0 to 3 as follows: no meeting participation = 0; meeting on as needed basis = 1; meeting every 2–3 months = 2; regularly attending monthly meetings = 3;^6^ hospital participation in Breaking Inequality Reimagining Transformative Healthcare (B.I.R.T.H.) Equity Maryland (Maryland Hospital Association and Maryland Patient Safety Center Equity Workgroup) in 2023;^7^ number of physicians in the hospital that interacted with MDMOM staff for any program component since October 1, 2022 (i.e., last 6 months);^8^ number of nursing staff in the hospital that interacted with MDMOM staff for any program component since October 1, 2022 (i.e., last 6 months);^9^ number of physicians and nurses in the hospital that interacted with MDMOM staff for any program component since October 1, 2022 (i.e., last 6 months);^10^ ranging from 1 to 4 for each hospital based on participation in four state groups: Birth Accountability Group; Morbidity, Mortality, and Quality Review Committee; Maternal Mortality Review Team; Maternal Health Task Force. ED, Emergency Department; MDMOM, Maryland Maternal Health Innovation Program; SMM, severe maternal morbidity.

Birthing hospitals represent our main implementation partners. The strength of their participation in MDMOM QI activities is positively correlated to their participation in check-in meetings and even more so with the degree of involvement by physicians in such activities (Fig. 4). We also find that higher engagement in MDMOM QI activities is positively correlated to hospitals’ participation in other maternal health initiatives coordinated by partner organizations as well as their being represented in state-level groups focusing on maternal health. Unfortunately, our data also show that hospitals with higher SMM rates tend to have lower engagement in MDMOM and other QI activities and lower physician and nurse involvement in such, but to be better represented in state-level groups that tackle maternal health issues.

## Discussion

MDMOM has had numerous successes, all of which were made possible through support of our partners and stakeholders. Currently, Maryland is the only US state to systematically use the specific guidance from professional organizations^16,34^ to conduct hospital-based surveillance for SMM. Key to the success of this surveillance program are the data abstractors and hospital review committee Chairs that champion this work—it is only with their support and commitment that the program continues and can inform future policy and programs to reduce SMM in Maryland. Statewide actors, MDH, MPSC, and MHA, request and use these data to inform their work across Maryland. The Equity Initiative has reached all 32 birthing hospitals in the state and trained a majority of the maternal health workforce in implicit bias. It will soon reach its second phase with the introduction of the Equity Toolkit, which will expand focus from individual provider interactions to system-level interventions to promote equity. The Telehealth Initiative is also gaining new supporters across the state. The state Perinatal/Neonatal Quality Collaborative is currently implementing American College of Obstetricians and Gynecologists/AIM’s severe hypertension bundle and our Telehealth Initiative serves as a useful complement. EMPOWER Moms materials are used by both community and hospital partners in innovative ways—printed warning sign education materials translated in 12 languages are shared in Emergency Departments, labor and delivery units, prenatal and postpartum clinics; videos are played in waiting rooms in various hospitals and FQHCs; and the online training originally developed for home visitors is currently being considered for training WIC, city and county health department staff.

MDMOM has encountered many challenges over the past 3+ years, especially given the COVID-19 pandemic. For example, most SMM data abstractors also have clinical responsibilities, which took priority over data abstraction, changes in unit leadership, staff turnover, funding uncertainties, and competing priorities, and QI activities were significant barriers to provider trainings. Although MDMOM has centered the design of the telehealth portfolio on the identified needs and preferences of end users, implementation has highlighted challenges in facilitating access given disparities in coverage, digital literacy, and access to technology. Furthermore, building trust with implementation partners and other collaborators took longer given COVID-19 restrictions on in-person meetings, with the consequence that implementation support was provided almost entirely remotely.

The program is innovative for several reasons. First, it involves a comprehensive set of statewide, data-driven, equity-centered, hospital, and community initiatives with limited evidence of effectiveness prior to the start of MDMOM. The program aims to not only increase awareness of key social, clinical, and health system contributors to adverse maternal outcomes (e.g., SMM surveillance, Equity Initiative online trainings) but to also propose ways to mitigate (e.g., SMM surveillance recommendations, Equity Initiative skill building sessions) and measure (e.g., Bias in Maternal Health Care scale) their influence. Importantly, for all activities, we rely on voluntary participation by target populations with a shared mental model for improving maternal health outcomes. Program activities have not been previously implemented at scale, and, of note, adaptation will be needed for their implementation elsewhere. Lastly, the program is designed to generate evidence of success, limitations, and challenges in real time, and to disseminate findings to a diverse audience through about 300 traditional and nontraditional partners and stakeholders, including the Task Force members.

The MDMOM program is not without limitations. We are yet to have patient representatives in hospital-based committees reviewing SMM events. Further increasing the number of perinatal health care providers taking our trainings depends on our ability to listen and, together with partners and the Task Force, to continue to build awareness and commitment to addressing health inequities in maternal health across the state. Many barriers exist to patients’ using telehealth and feeling empowered to follow-up with their obstetric providers when a problem, real or perceived, appears during pregnancy or postpartum. We need to use implementation science to better understand how to facilitate the adoption of telehealth interventions especially among socioeconomically disadvantaged populations. Finally, only about half of home-visiting and community-based organizations offering services to pregnant and postpartum families in Maryland communities participated in the EMPOWER Moms trainings and community of practice network.

Because of the complexity of the maternal health landscape and the magnitude of maternal health inequities in Maryland, MDMOM was intentionally designed to include diverse stakeholders—this is evidenced by the composition of the Task Force we established in the spring of 2020. Moreover, our activities articulate an ongoing, comprehensive learning process in which equity is operationalized collectively and intentionally over time, using available data. The main lessons learned to date include the need to: (1) build awareness, commitment, and a shared model to addressing health equity within and between partners and stakeholders, (2) dedicate time to learn about the work done by other organizations (i.e., state partners, community-based, and academic centers) working in the maternal health space, so to avoid duplication of efforts and promote resource sharing, (3) aim to gain a deep knowledge of the context and seek to identify not only potential barriers to program implementation but also facilitators to overcome those barriers, (4) when failure is more likely than success (e.g., situation encountered while working on training and telemedicine interventions in 2020), use a “fail fast” mentality and change course, and (5) strive for direct, honest, and continuous communications, making time for authentic dialogue to develop a shared language, mission, and vision for the program being implemented.

## Conclusion

Our experience with the MDMOM program demonstrates that an equity focus and broad stakeholder collaboration building strong relationships and providing implementation support can lead to high levels of engagement in innovative maternal health interventions.

## Supplementary Material

Supplementary Table S1

## Abbreviations Used

AIM: Alliance for Innovation on Maternal Health
BHS: Baltimore Healthy Start
B.I.R.T.H.: Breaking Inequality Reimagining Transformative Healthcare
CDC: U.S. Centers for Disease Control and Prevention
EMPOWER: Enhanced Maternal and Postpartum Warning signs Education and Recognition
FQHC: Federally Qualified Health Centers
HRSA: Health Resources and Services Administration
MDH: Maryland Department of Health
MDMOM: Maryland Maternal Health Innovation Program
MHA: Maryland Hospital Association
MPSC: Maryland Patient Safety Center
QI: quality improvement
SMM: severe maternal morbidity
SUD: substance use disorder
