# Supplementary material for: Centering Equity and Fostering Stakeholder Collaboration and Trust—Pillars of the Maternal Health Innovation Program in Maryland
Source: Health Equity. 2024 Jun 27;8(1):406–18. doi: 10.1089/heq.2023.0127 (PMC11249133; doi:10.1089/heq.2023.0127)
Supplement: Supplementary Table S1 [file heq.2023.0127_etable_submitted.docx]

e-Table 1. Hospital engagement in MDMOM and other state maternal health activities

| **Level of care** | **2021 deliveries** | **Equity Initiative^1^** | **Telehealth Initiative^2^** | **SMM Surveillance^3^** | **Use of EMPOWER Moms materials^4^** | **MDMOM meeting participation^5^** | **Physician involvement^6^** | | **Nursing involvement^6^** | | **Emergency Department Involvement^7^** | **Representation in key state-level committees^8^** |
| --- | --- | --- | --- | --- | --- | --- | --- | --- | --- | --- | --- | --- |
|  |  |  |  |  |  |  | *Physician Leadership* | *Other Physician* | *Nursing Leadership* | *Other Nursing* |  |  |
| IV | 2382 | 2 | 1 | 3 | 2 | 1* | 1 | 0 | 1 | 2 | 0 | MMRT, TF, MMQRC, AG |
| IV | 1778 | 2 | 1 | 3 | 0 | 2* | 0 | 0 | 2 | 1 | 0 | MMRT, TF, MMQRC, AG |
| III | 3971 | 3 | 1 | 3 | 1 | 3* | 1 | 1 | 3 | 3 | 1 | AG |
| III | 5542 | 4 | 2 | 3 | 1 | 3 | 0 | 2 | 4 | 2 | 0 | TF, MMQRC. AG |
| III | 1472 | 4 | 1 | 0 | 2 | 3 | 1 | 0 | 2 | 0 | 1 | MMRT, AG |
| III | 2508 | 3 | 1 | 0 | 1 | 2 | 0 | 0 | 2 | 0 | 1 | AG |
| III | 4327 | 4 | 3 | 1 | 1 | 3 | 0 | 0 | 1 | 0 | 1 | AG |
| III | 8723 | 1 | 0 | 2 | 2 | 2 | 0 | 0 | 1 | 0 | 0 | MMRT, TF, AG |
| III | 2619 | 4 | 3 | 3 | 1 | 3* | 0 | 2 | 3 | 0 | 0 | -- |
| III | 1270 | 3 | 2 | 3 | 1 | 3* | 1 | 0 | 1 | 2 | 0 | -- |
| III | 2174 | 2 | 3 | 1 | 1 | 2 | 1 | 0 | 3 | 2 | 0 | AG |
| III | 2575 | 4 | 3 | 3 | 1 | 2* | 1 | 1 | 0 | 0 | 1 | MMRT, TF, AG |
| III | 1758 | 3 | 3 | 3 | 1 | 2* | 1 | 0 | 1 | 1 | 1 | MMRT |
| III | 1230 | 2 | 3 | 2 | 1 | 0 | 0 | 1 | 2 | 1 | 0 | -- |
| III | 1741 | 3 | 0 | 0 | 2 | 2 | 0 | 0 | 1 | 1 | 0 | MMRT, TF |
| II | 1257 | 2 | 2 | 0 | 2 | 2 | 0 | 0 | 1 | 0 | 1 | AG |
| II | 1052 | 4 | 3 | 3 | 1 | 3 | 0 | 0 | 1 | 3 | 1 | AG |
| II | 1772 | 3 | 0 | 2 | 1 | 3 | 0 | 0 | 1 | 1 | 0 | AG |
| II | 1253 | 3 | 3 | 0 | 1 | 2 | 1 | 0 | 3 | 1 | 0 | AG |
| II | 581 | 2 | 0 | 0 | 0 | 3 | 0 | 0 | 2 | 2 | 1 | AG |
| II | 927 | 2 | 0 | 0 | 0 | 1 | 1 | 0 | 1 | 0 | 0 | AG |
| II | 1738 | 1 | 1 | 0 | 1 | 0 | 0 | 0 | 2 | 0 | 1 | AG |
| II | 1928 | 1 | 2 | 0 | 1 | 1 | 0 | 0 | 2 | 0 | 0 | TF, AG |
| II | 1590 | 2 | 3 | 0 | 1 | 2 | 1 | 0 | 4 | 1 | 1 | MMRT |
| II | 1332 | 2 | 0 | 0 | 0 | 1 | 1 | 0 | 1 | 0 | 0 | MMRT |
| II | 823 | 2 | 3 | 3 | 2 | 1* | 1 | 0 | 1 | 2 | 0 | TF, AG |
| I | 654 | 2 | 2 | 3 | 1 | 1 | 1 | 0 | 1 | 0 | 0 | AG |
| I | 246 | 1 | 1 | 0 | 0 | 2 | 1 | 0 | 1 | 0 | 0 | AG |
| I | 1110 | 2 | 3 | 3 | 1 | 2 | 1 | 1 | 1 | 1 | 0 | MMQRC, AG |
| I | 529 | 3 | 2 | 0 | 1 | 3 | 0 | 0 | 1 | 1 | 0 | -- |
| I | 1007 | 3 | 2 | 1 | 1 | 2 | 0 | 0 | 2 | 0 | 0 | MMRT |
| I | 401 | 4 | 1 | 0 | 1 | 2 | 0 | 0 | 1 | 0 | 0 | AG |

*Notes:* ^1^Participation in 1 to 4 types of trainings; ^2^ No interest=0; only interest but no activity=1; distributing BP cuffs or referring to Optum=2; distributing BP cuffs and referring to Optum=3; ^3^ No interest=0; only interest but no activity=1; entering data=2; timely complete case data submission defined as quarterly or more frequent review of cases-3; ^4^ no utilization of EMPOWER Moms materials in hospitals=0; distribution of warning signs handouts=1; innovative use of materials in hospitals=2; ^5^ No meeting participation=0; meeting on as needed basis=1; meeting every 2-3 months=2; regularly attending monthly meetings=3 & * added for hospitals participating in SMM surveillance and attending most (>50%) learning & sharing meetings; ^6^ Number of physicians/nurses that interacted with MDMOM staff for any program component since Oct 1, 2022 (i.e., last 6 months); ^7^ participation in B.I.R.T.H. Equity Maryland (Maryland Hospital Association and Maryland Patient Safety Center Equity Workgroup); ^8^ As of April 1, 2023.

AG, Birth Accountability Group; MMQRC, Morbidity, Mortality, and Quality Review Committee; MMRT, Maternal Mortality Review Team; TF, Maternal Health Task Force.
